# Impact of estrogen deficiency on diaphragm and leg muscle contractile function in female *mdx* mice

**DOI:** 10.1371/journal.pone.0249472

**Published:** 2021-03-31

**Authors:** Pangdra Vang, Cory W. Baumann, Rebecca Barok, Alexie A. Larson, Brendan J. Dougherty, Dawn A. Lowe

**Affiliations:** 1 Division of Rehabilitation Science, Department of Rehabilitation Medicine, University of Minnesota, Minneapolis, Minnesota, United States of America; 2 Department of Integrative Biology and Physiology, University of Minnesota, Minneapolis, Minnesota, United States of America; 3 Division of Physical Therapy, Department of Rehabilitation Medicine, University of Minnesota, Minneapolis, Minnesota, United States of America; Universitat Wien, AUSTRIA

## Abstract

Female carriers of Duchenne muscular dystrophy (DMD) presenting with DMD symptomology similar to males with DMD, such as skeletal muscle weakness and cardiomyopathy, are termed manifesting carriers. There is phenotypic variability among manifesting carriers including the age of onset, which can range from the first to fourth decade of life. In females, estrogen levels typically begin to decline during the fourth decade of life and estrogen deficiency contributes to loss of muscle strength and recovery of strength following injury. Thus, we questioned whether the decline of estrogen impacts the development of DMD symptoms in females. To address this question, we studied 6–8 month-old homozygous *mdx* female mice randomly assigned to a sham or ovariectomy (OVX) surgical group. *In vivo* whole-body plethysmography assessed ventilatory function and diaphragm muscle strength was measured *in vitro* before and after fatigue. Anterior crural muscles were analyzed *in vivo* for contractile function, fatigue, and in response to eccentric contraction (ECC)-induced injury. For the latter, 50 maximal ECCs were performed by the anterior crural muscles to induce injury. Body mass, uterine mass, hypoxia-hypercapnia ventilatory response, and fatigue index were analyzed by a pooled unpaired t-test. A two-way ANOVA was used to analyze ventilatory measurements. Fatigue and ECC-injury recovery experiments were analyzed by a two-way repeated-measures ANOVA. Results show no differences between sham and OVX *mdx* mice in ventilatory function, strength, or recovery of strength after fatigue in the diaphragm muscle or anterior crural muscles (p ≥ 0.078). However, OVX mice had significantly greater eccentric torque loss and blunted recovery of strength after ECC-induced injury compared to sham mice (p ≤ 0.019). Although the results show that loss of estrogen has minimal impact on skeletal muscle contractile function in female *mdx* mice, a key finding suggests that estrogen is important in muscle recovery in female *mdx* mice after injury.

## Introduction

Duchenne muscular dystrophy (DMD) is a devastating muscular dystrophy, affecting approximately 1 in 5,000 male births, where a complete absence of dystrophin causes progressive muscle degeneration and weakness, ultimately leading to death from respiratory or cardiac failure [1–4]. Despite DMD occurring predominantly in males, female carriers (i.e., females who carry a mutation of the *DMD* gene on one of their X-chromosomes) may also display symptoms of DMD [5,6]. Women displaying symptoms are referred to as manifesting carriers [5,6]. Moser and Emery cites the first case of a manifesting carrier of DMD was in 1934 by Kryschowa and Abowjan [6]. While the majority of female carriers do not manifest symptoms, studies have shown that 2–17% develop DMD symptomology [5,7,8]. Similar to males with DMD, manifesting carriers present with skeletal muscle weakness and cardiomyopathy, with the gravity of skeletal muscle weakness ranging from mild muscle weakness to more severe DMD-like progression [8–11].

Literature on female carriers and manifesting carriers of DMD is comprised of cohort studies, case studies, and meta-analyses, focused on X-chromosome inactivation and phenotype characterization with the majority of information on cardiomyopathy and limited information on skeletal muscle weakness [5,7,8,12–17]. The vast majority of research using rodent models of DMD, such as the commonly used *mdx* mouse, has used males to investigate the pathogenesis of and treatments for DMD, with literature on DMD signs in females being sparse [18]. There is a paucity of information on female and manifesting carriers in both human and rodent literature, particularly in examining skeletal muscle weakness. Previous studies on manifesting carriers investigating skeletal muscle weakness reported that the age of onset varied from the first decade into the fourth decade of life and that the weakness may progress with age [8,14,16]. As this broad time to symptom development in manifesting carriers overlaps the natural decline in female estrogen levels, we questioned whether symptom development and estrogen levels were related in females lacking dystrophin.

Reduction of circulating estrogen in females begins around the fourth decade of life occurring as a natural biological process concluding with menopause. However, estrogen deficiency can also be triggered by other events [19–26]. There is evidence in women that the loss of estrogen contributes to muscle weakness and reduced performance [27,28] and that estrogen-based hormone therapy mitigates the progression of weakness [29,30]. Likewise, some but not all studies in female rodents have shown that estrogen deficiency contributes to skeletal muscle weakness and can be reversed or prevented by treatment with 17-β estradiol (E2) [31]. Furthermore, estrogen deficiency is associated with reduced recovery of strength after muscle injury [32–35]. The recovery of strength following injury is particularly important in dystrophic muscle because the lack of dystrophin increases susceptibility of muscle to contraction-induced injury and subsequent muscle weakness [36–38].

Here, we hypothesized that estrogen deficiency would decrease skeletal muscle strength and diminish the capacity of muscle to recover from injury in female *mdx* mice. Using an ovariectomy model to reduce ovarian hormone production, the major hormone being estradiol, we used three approaches to evaluate the impact of hormone deficiency on skeletal muscle of female *mdx* mice, 1) assessment of ventilatory function, 2) assessment of contractile function in the diaphragm and anterior crural muscles, and 3) assessment of strength recovery after fatigue and injury. Elucidating the effects of estrogen on skeletal muscle strength and recovery in female *mdx* mice may aid in determining interventional therapies to mitigate skeletal muscle weakness in manifesting carriers of DMD, particularly as these women age, are potentially under the stress of caregiving, and endure estrogen declines.

## Methods and experimental design

### Animals

Homozygous female *mdx* mice were bred on-site with breeders from Jackson Laboratories (Bar Harbor, ME, USA), and experiments began when mice were 6 to 8 months of age (n = 24). Mice were housed in groups of three to five and had access to phytoestrogen-free food and water ad libitum. The room was maintained on a 14:10 h light/dark cycle. The Institutional Animal Care and Use Committee at the University of Minnesota approved all animal procedures.

### Experimental design

**Fig 1:**
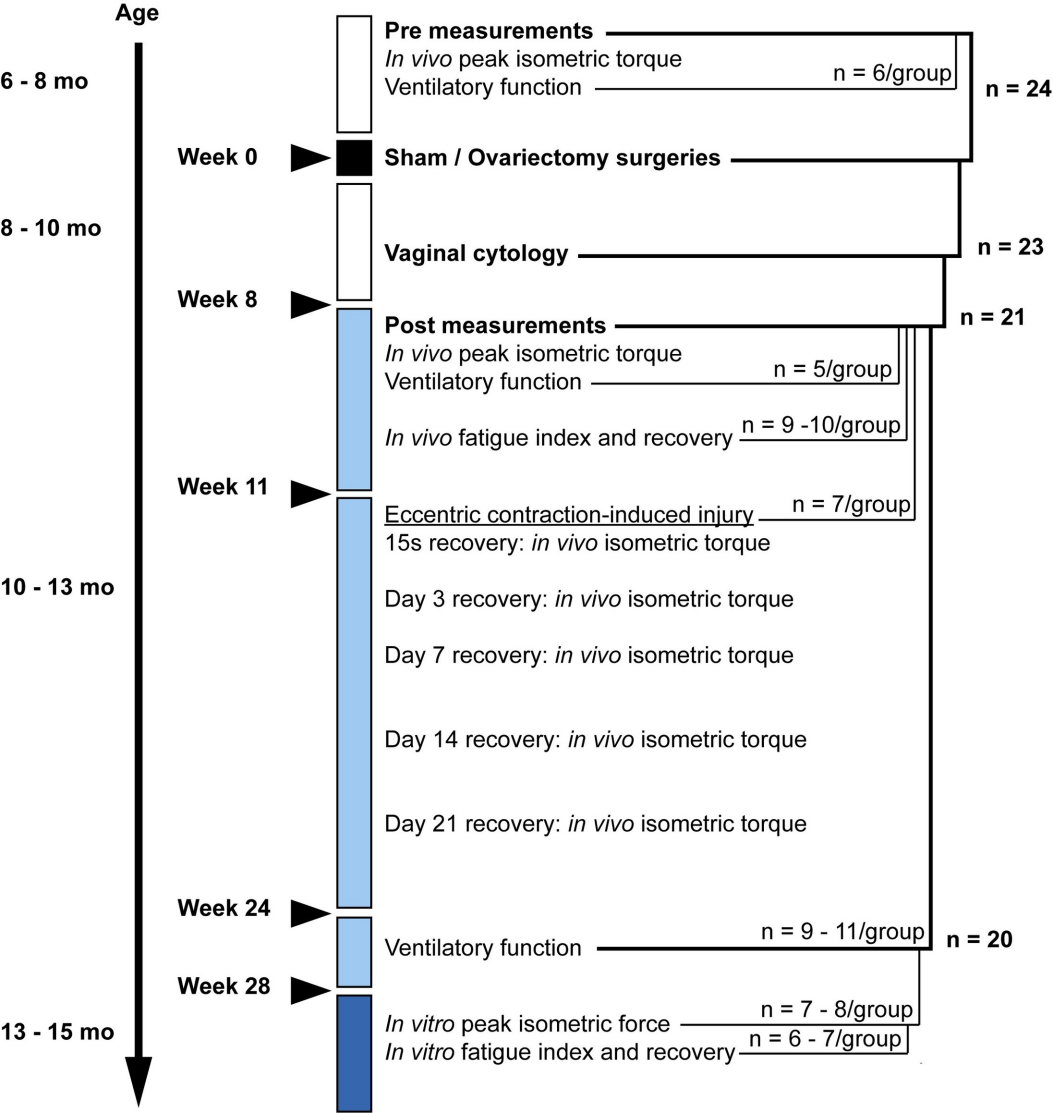
Schematic of experimental design. A total of 24 mice were used with subsets used for various measurements as indicated by n per group. The black square represents week 0, when surgeries were performed. All weeks listed are relative to those post-surgery. White and light blue boxes represent pre and post *in vivo* measurements performed on the anterior crural muscles and respiratory muscles. The dark blue box represents *in vitro* diaphragm measurements that were made over the course of two weeks. Depicted at the right side is the age range of the mice during each segment of the study. Horizontal text describes the major interventions and measurements and the times during the study when they were completed.

The study design to assess skeletal muscle contractile function of the diaphragm and anterior crural muscles (i.e., tibialis anterior (TA), extensor digitorum longus (EDL), extensor hallucis longus, and peroneus tertius) is summarized in Fig 1. *In vivo* “Pre” maximal isometric torque of the anterior crural muscles and ventilatory function of the respiratory muscles were performed at the start of the study to determine baseline function (n = 24). Female *mdx* mice, matched-based on maximal isometric torque, were then randomly assigned to a sham or ovariectomy (OVX) group and underwent respective surgeries. Vaginal cytology was performed 6 weeks post-surgeries for 4 consecutive days to verify estrous cycling in sham mice and successful ovariectomy in OVX mice (37). During the study, a total of 4 mice died: 1 mouse was euthanized with CO_2_ after surgery due to sickness behavior (i.e., immobility, inactivity, reduction of grooming, and reduced intake of food and water) and 3 died of unknown causes (Fig 1 provides subsets of n’s for each phase of the study, with total n = 20 at the end of the study shown). For some tests, investigators were blinded but it was not possible for each experiment.

Eight to ten weeks after surgeries, *in vivo* “Post” measurements of ventilatory function and anterior crural muscle torque and fatigability were measured (n = 5/group and 8–10/group, respectively). A week later, the anterior crural muscles were assessed for torque again and then performed a series of eccentric contractions to induce injury (n = 7/group). Recovery of maximal isometric torque from ECC-induced injury was measured over the next three weeks. Near the end point of the study, *in vivo* ventilatory function was re-measured (n = 9–11/group) followed by *in vitro* contractile testing of the diaphragm including measurements of force, fatigue, and recovery of force after fatigue (n = 6–8/group). As a second confirmation of successful surgeries, at the end point, uteri were dissected and weighed.

### Sham and OVX surgeries

Sham and OVX surgeries were done as previously described [39]. Briefly, mice were anesthetized (1.75% isoflurane and 200 ml O_2_ per min) and slow-release buprenorphine (1 mg/kg) was delivered subcutaneously. The females in the OVX group had bilateral ovariectomy and those in the sham group had sham ovariectomy (i.e. bilateral abdominal incisions and then sutured without the removal of the ovaries. The abdominal incisions were closed with 6–0 silk sutures and the skin incision with 7 mm wound clips.

### Whole-body plethysmography

Awake, unrestrained female *mdx* mice were recorded during two ventilatory behaviors: spontaneous breathing of room air (eupnea) and spontaneous breathing challenged by hypoxia–hypercapnia (HH; 10% O_2_, 5% CO_2,_ balanced N_2_) using a whole-body plethysmography (WBP) system (Data Sciences International, St. Paul, MN, USA). Mice were weighed and placed into one of two plethysmograph chambers with recordings done in parallel and groups (sham, OVX) counterbalanced. WBP recordings and analysis were adapted from previously described [40–42]. Briefly, all mice were acclimated for 10 min, followed by a 45 min recording session during eupnea, and 5 min recording session during HH for a total duration of 1 hr. Data collection of the ventilatory parameters respiratory rate (RR), tidal volume (V_T_), and minute ventilation (V_E_) were analyzed for a 2 min period of eupnea and a 2 min period of HH using Ponemah 5 (Data Sciences International). V_T_ and V_E_ were normalized to mouse body weight and expressed as ml/g and ml/g/min^-1^, respectively. The duty cycle of the diaphragm, expressed as a percentage, was calculated as: $duty\;cycle=(\frac{T_{i}}{T_{tot}})*100$, where T_i_ represents inspiratory time, and T_tot_ represents total time (T_i_ + T_e_ [expiratory time]). The HH ventilatory response examining V_E_ response to HH was determined as a percent change from eupnea: $HH\;ventilatory\;response=(\frac{eupnea-HH}{eupnea})*100$.

### *In vitro* diaphragm contractility

#### Maximal and submaximal isometric forces

Mice were anesthetized with a sublethal dose of pentobarbital (100 mg/kg) and the diaphragm muscle was dissected and immediately placed in a petri dish that was lined with ~8 mm of black sylgard (DD-90-S-BLK, Living Systems Instrumentation, St. Albans City, VT) and filled with oxygenated (95:5% O_2_:CO_2_) Krebs-Ringer bicarbonate buffer (pH 7.4). The diaphragm was pinned with stainless steel minuten pins and two diaphragm strips (~3–4 mm wide) from the midcostal regions were excised with the central tendon and ribcage attached [43,44]. The central tendon and ribcage of the diaphragm strip was tied with 6–0 silk sutures, and then it was placed in an organ bath, filled with 1.5 ml of Krebs-Ringer bicarbonate buffer maintained at 25°C. The suture on the central tendon was attached to a dual-mode muscle lever system (300B-LR; Aurora Scientific Aurora, ON, Canada) and the suture on the ribcage secured to the base of the organ bath. Measurement of maximal isometric force was performed from previously described methods [44]. Briefly, diaphragm muscle strips were acclimated for 10 min in the bath before performing isometric twitch contractions at 150 V via platinum electrodes with 1 min of rest between contractions. Length adjustments were made to obtain maximal twitch force (P_t_) and then optimal length (L_o_) was measured. A force-frequency protocol was performed in which 10 isometric contractions were performed at 5, 10, 20, 30, 40, 50, 60, 75, 85, and 100 Hz for 500 ms with 0.5 ms pulses and 2 min rest between contractions. The 100 Hz stimulation was used to describe maximal isometric tetanic force (P_o_). Specific isometric tetanic force (sP_o_) was calculated by normalizing P_o_ to the muscle strip cross-sectional area (CSA): $CSA=\frac{muscle\;mass}{\left(Lo*muscle\;density\right)}$.

*Fatigue and recovery*. To assess fatigability and recovery of strength after fatigue, the diaphragm muscle strip performed 120 submaximal isometric contractions (40 Hz for 330 ms with 0.5 ms pulses) every s over a 2 min period. A fatigue index (FI) [51] assessing fatigue resistance was calculated as the ratio of the final force (FF) measured after 2 min of 120 submaximal contractions to the initial force (IF): $FI=(\frac{FF}{IF})*100$. Fatigue recovery was assessed by determining P_o_ at 15 s (0 min), 2 min, 4 min, and 6 min after the fatigue protocol. The muscle was then removed from the bath, blotted, and weighed.

### *In vivo* contractile function and injury of the anterior crural muscles

#### Maximal isometric torque

To determined maximal isometric torque, anesthetized mice (1.25% isoflurane and 125 ml O_2_ per min) were placed on a temperature-controlled platform to maintain core body temperature between 35°C and 37°C [45,46]. The left knee was immobilized and the left foot secured to an aluminum “shoe” attached to the shaft of an Aurora Scientific 300B servomotor. Sterilized platinum needle electrodes (FE212, Grass Technologies Warwick, RI, USA) were inserted through the skin to specifically stimulate the common peroneal nerve of the left leg. Stimulation voltage and needle electrode placement were optimized with 10–20 isometric contractions (250 Hz for 150 ms with 0.1 ms pulses). Following optimization, maximal isometric torque was measured before the fatigue and injury protocols. All torque measurements were normalized to mouse body weight and expressed as mN·m·kg^-1^.

#### Fatigue and recovery

To assess fatigability and recovery of strength after fatigue, the anterior crural muscles were repeatedly stimulated with 120 submaximal isometric contractions (40 Hz for 330 ms with 0.1 ms pulses) every s over a 2 min period [47]. Fatigue recovery was assessed by determining isometric torque at 15 s (0 min), 1 min, 3 min, 5 min, 7 min, and 10 min after the fatigue protocol. FI was calculated as described for the diaphragm muscle.

#### ECC-induced injury

ECC-induced injury was performed as previously described [48]. In brief, anesthetized mice (1.25% isoflurane and 125 ml O_2_ per min) were maintained on the temperature-controlled platform with the left positioned as described for maximal isometric torque and 50 maximal ECCs were performed by the anterior crural muscles. For each ECC, the left foot was passively rotated about the ankle from 0° (i.e., when the foot is perpendicular to the tibia) to 19° dorsiflexion, followed by 38° of plantarflexion at 2000°/s while being stimulated (250 Hz for 100 ms with 0.1 ms pulses) with 10 s of rest between contractions. Maximal isometric torque was measured 15 s after the last contraction (0 min), and recovery of maximal isometric torque was measured 3, 7, 10, 14, and 21 d post-injury.

### Statistical analysis

Data were analyzed using Graphpad Prism 8 (San Diego, CA, USA). Ventilatory parameters were analyzed using two-way ANOVA with group (sham vs. OVX) and time (pre vs. 8 wk vs. 24 wk) or behavior (eupena vs. HH) as grouping variables. Body mass, uterine mass, hypoxia-hypercapnia ventilatory response, and FI data were analyzed using a pooled unpaired t-test (sham vs. OVX). Data from the fatigue and ECC-injury recovery experiments were analyzed by a two-way repeated-measures ANOVA, with group and contraction number or time as grouping variables. Bonferroni’s *post-hoc* analysis was conducted when main effects or interactions were detected. All data are reported as the mean ± standard error of the mean (SEM). Significance was accepted at α < 0.05 level.

## Results

**Fig 2:**
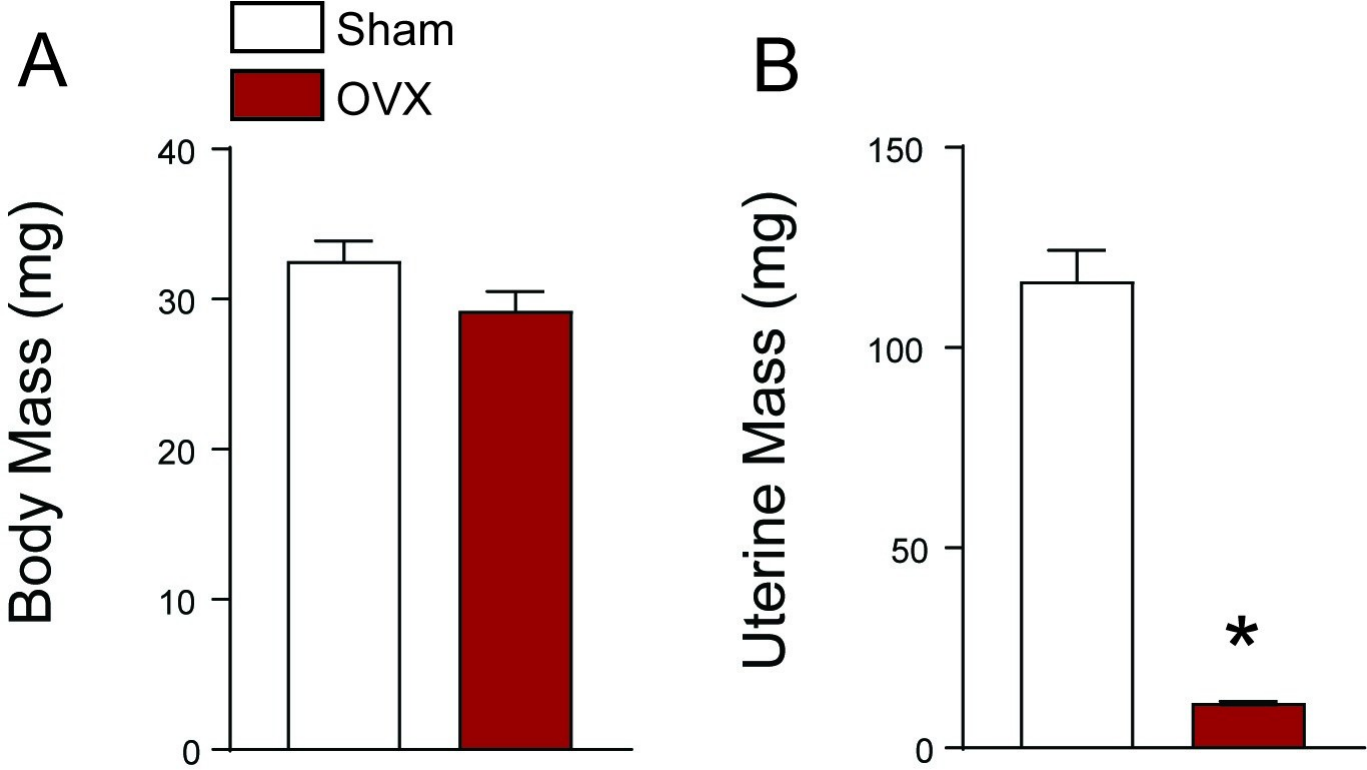
Body and uterine masses of female *mdx* mice with and with ovaries. (A) Body mass measured at terminal diaphragm *in vitro* experiment (p = 0.095). (B) Uterine mass also measured at the terminal experiment (p < 0.001). Data were analyzed by a pooled t-test (Sham vs. OVX); n = 7–8 per group. Values represent means ± SEM. *Significantly different from sham.

Vaginal cytology confirmed estrous cycling in sham *mdx* mice and persistent diestrus in OVX *mdx* mice [34]. There was no significant difference in body mass between sham and OVX mice at the end of the study (p = 0.095; Fig 2A). As expected, uterine mass was significantly different between groups (p < 0.001; Fig 2B). The mean uterine masses for sham and OVX female *mdx* mice were 116.6 ± 7.8 mg and 11.3 ± 0.4 mg, respectively, confirming successful removal of ovarian tissue.

### Estrogen deficiency and ventilatory function in female *mdx* mice

Ventilatory function changes across time during eupnea and HH are shown in Table 1. There were no significant changes in RR, V_T_, and V_E_, across time (Pre, 8, and 24 wk) or between sham and OVX mice during eupnea (p ≥ 0.163) except for duty cycle, which was ~20% higher at 8 and 24 wk than Pre. During HH, a main effect of time was detected for all ventilatory parameters, in general with those at later time points being lower than at Pre (p ≤ 0.028). There was no difference between sham and OVX mice for ventilatory measures during HH.

**Table 1:** Ventilatory function in awake female *mdx* mice during whole body plethysmography.

**Eupnea**									
**Time Pre/Post-surgery**	**Pre**		**8 wk**		**24 wk**		**Two—way ANOVA (p-values)**		
**Group**	**Sham (6)**	**OVX (6)**	**Sham (5)**	**OVX (5)**	**Sham (9)**	**OVX (11)**	**Group**	**Time**	**Interaction**
**RR (min**^**-1**^**)**	227 ± 26	213 ± 24	238 ± 20	215 ± 19	254 ± 20	224 ± 8	0.163	0.534	0.917
**V**_**T**_ **(ml/g)**	0.016 ± 0.001	0.016 ± 0.001	0.016 ± 0.002	0.017 ± 0.002	0.016 ± 0.001	0.017 ± 0.001	0.293	0.899	0.836
**V**_**E**_ **(ml/g/min**^**-1**^**)**	4.4 ± 0.5	3.9 ± 0.4	4.3 ± 0.4	3.9 ± 0.4	4.5 ± 0.3	4.2 ± 0.2	0.228	0.802	0.966
**Duty cycle (%)**	30.4 ± 1.9	31.2 ± 1.3	38.2 ± 3.2	36.2 ± 2.0	38.1 ± 2.1	36.3 ± 1.2	0.541	0.003	0.731
**Hypoxia—hypercapnia**									
**Time Pre/Post-surgery**	**Pre**		**8 wk**		**24 wk**		**Two—way ANOVA (p-values)**		
**Group**	**Sham (6)**	**OVX (6)**	**Sham (5)**	**OVX (5)**	**Sham (9)**	**OVX (11)**	**Group**	**Time**	**Interaction**
**RR (min**^**-1**^**)**	332 ± 10	327 ± 7	265 ± 12	274 ± 11	274 ± 11	295 ± 10	0.374	<0.001	0.482
**V**_**T**_ **(ml/g)**	0.024 ± 0.001	0.025 ± 0.002	0.022 ± 0.002	0.021 ± 0.002	0.019 ± 0.001	0.021 ± 0.001	0.615	0.028	0.698
**V**_**E**_ **(ml/g/min**^**-1**^**)**	8.0 ± 0.4	8.2 ± 0.6	6.0 ± 0.5	5.8 ± 0.6	5.5 ± 0.5	6.4 ± 0.4	0.511	<0.001	0.557
**Duty cycle (%)**	44.3 ± 0.2	44.2 ± 0.3	35.4 ± 1.6	34.6 ± 1.0	43.5 ± 2.0	42.3 ± 1.6	0.626	<0.001	0.937

Data are mean ± SEM. RR = respiratory rate, V_T_ = tidal volume, V_E_ = minute ventilation.

**Fig 3:**
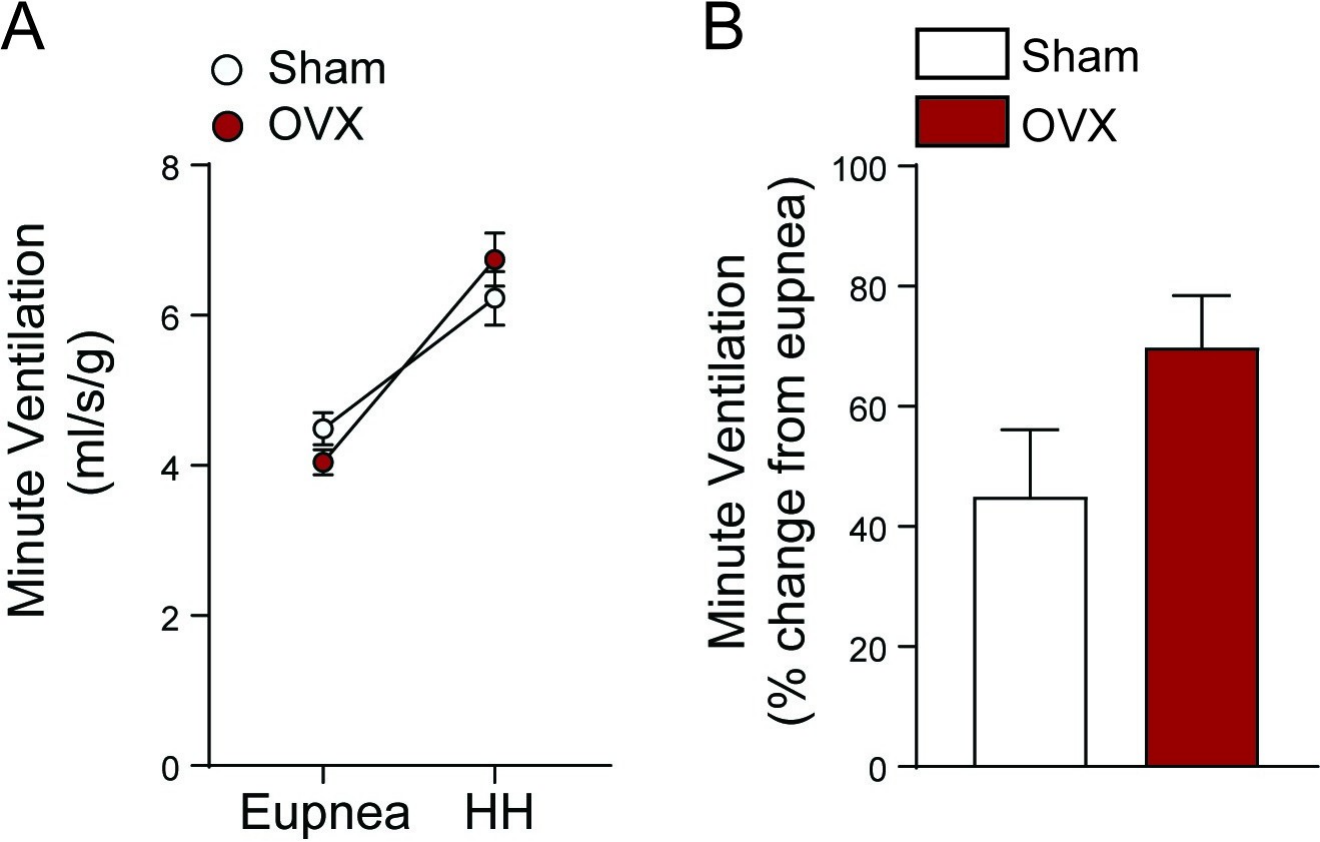
V_E_ changes across behaviors and hypoxia-hypercapnia ventilatory response in female *mdx* mice. V_E_ across all timepoints (Pre, 8, and 24 wks) was pooled to examine V_E_ across behaviors and the hypoxia-hypercapnia ventilatory response. A) V_E_ during eupnea and HH challenge in Sham and OVX female *mdx* mice. (B) Hypoxia-hypercapnia ventilatory response presented as change from eupnea. Data was analyzed by a two-way ANOVA for V_E_ across behaviors and groups: A main effect of behavior on V_E_ was found (p < 0.001), but no effect of group or group × behavior interaction was detected (p ≥ 0.0966). The hypoxia-hypercapnia ventilatory response was analyzed by a pooled unpaired t-test (p = 0.082). Values represent means ± SEM; n = 5–11 per group.

Examining V_E_ response across the behaviors showed the expected effect that V_E_ was greater during the HH challenge than during eupnea (p < 0.001; Fig 3A); however, there was no difference between sham and OVX mice for either behavior (p ≥ 0.903) and no effect of V_E_ on the group × behavior interaction (p = 0.097). Expressing V_E_ from the HH challenge as % change from eupnea also showed no significant difference between sham and OVX *mdx* mice (p = 0.082; Fig 3B). Because only effects of time were detected across the ventilatory parameters, these results indicate that estrogen deficiency has no impact on ventilatory function of adult female *mdx* mice.

### Estrogen deficiency and skeletal muscle contractility

**Fig 4:**
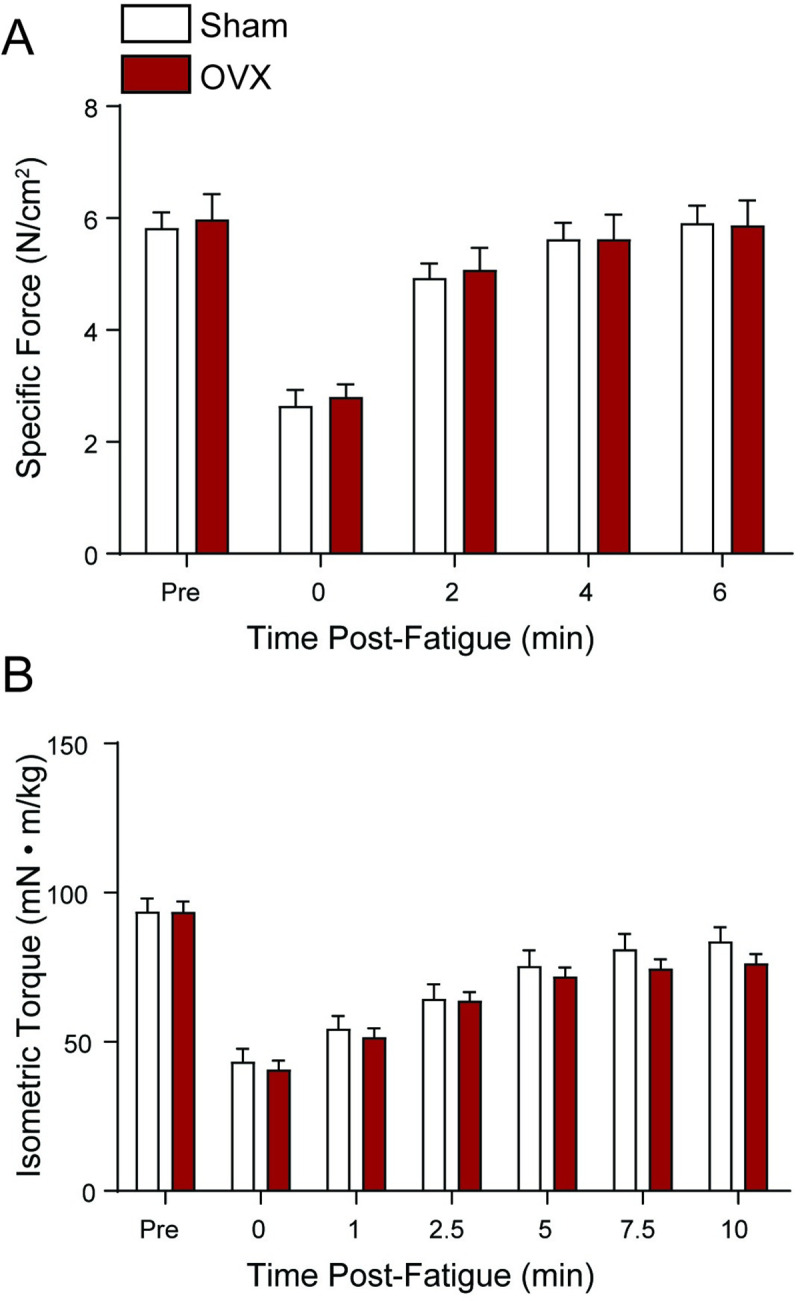
Strength and recovery of strength following submaximal fatiguing contractions. (A) Loss and recovery of isometric tetanic specific force in diaphragm muscle after a 2 min fatigue protocol in Sham and OVX *mdx* mice. (B) Anterior crural muscles’ recovery of isometric tetanic torque after a 2 min fatigue protocol in Sham and OVX *mdx* mice. Data were analyzed by two-way repeated-measures ANOVA. As designed, a main effect of time was measured for both diaphragm and anterior crural muscles (p < 0.001), but no effect of group or group × time was detected (p ≥ 0.201). Values represent means ± SEM; n = 6–7 per group.

Sham and OVX female *mdx* mice did not differ in diaphragm maximal specific force (p = 0.672; Fig 4A Pre values). The mean maximal sP_o_ in the diaphragm for sham and OVX mice were 5.9 ± 0.3 N/cm^2^ and 6.1 ± 0.4 N/cm^2^, respectively. Recovery of strength measured after the fatigue protocol showed no difference in sP_o_ of the diaphragm muscle between groups (p = 0.868) or group × time interactions (p = 0.876), with an effect of time demonstrating significant differences in strength during recovery (p < 0.001; Fig 4A). Likewise, the FI showed no effect of group (sham vs. OVX) in the diaphragm muscle (64.4 ± 2.5% and 61.1 ± 1.0%, respectively; p = 0.226).

Maximal isometric torque of the anterior crural muscles did not differ between sham and OVX mice (96.2 ± 4.6 and 91.8 ± 3.6 mN•m/kg body mass, respectively; p = 0.450; Fig 4B Pre values). Recovery of torque measured after the fatigue protocol showed a significant effect of time (p < 0.001) but no effect of group (p = 0.516) or group × time interaction (p = 0.201). Similarly, the FI did not differ between anterior crural muscles from sham and OVX mice (40.7 ± 3.7% and 30.3 ± 5.0%, respectively; p = 0.131). Overall, investigation of skeletal muscle contractile function through measurements of strength, fatigability, and recovery of strength after fatigue in both the diaphragm and anterior crural muscles did not reveal differences between sham and OVX mice. These results indicate that estrogen deficiency for up to 24 weeks does not decrease skeletal muscle strength in female *mdx* mice.

### Estrogen deficiency and recovery of strength after injury

**Fig 5:**
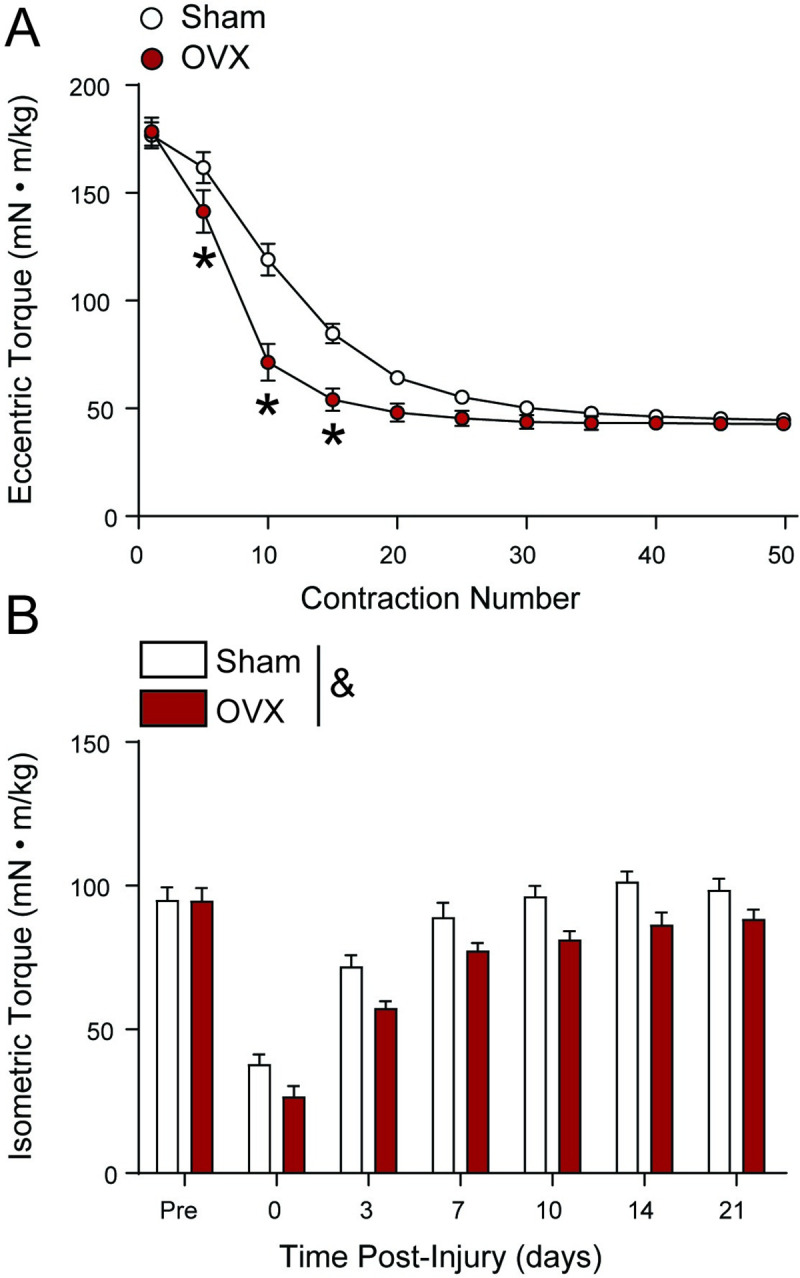
Estrogen deficiency accentuates eccentric force loss and blunts muscle recovery of force after ECC -induced injury. (A) Eccentric torque of the anterior crural muscles during 50 ECC designed to induce injury with data from every 5^th^ contraction plotted. (B) Recovery of isometric torque after ECC-induced injury. Data were analyzed by two-way repeated-measures ANOVA. In A, a group × contraction number interaction was detected followed by Bonferroni’s multiple comparisons test. *Significantly different from Sham at given contraction. In B, no group × time interaction was detected (p = 0.242) and as designed, there was a significant main effect time (p < 0.001). There was also a significant main effect of group (p = 0.006) with the OVX mice recovering less torque than Sham mice (denoted by &). Values represent means ± SEM; n = 7–8 per group.

The anterior crural muscles were challenged with an ECC protocol to induce injury and determine if estrogen deficiency diminished the capacity of muscles to recover strength. ECC torque during the protocol declined differently within the sham and OVX groups (group × contraction number interaction, p < 0.001; Fig 5A). *Post-hoc* analysis revealed that the OVX female *mdx* mice had significantly lower eccentric torque at contractions 5, 10, and 15 compared to sham. In general, regardless of estrogen level, both groups lost ~75% of eccentric torque in the anterior crural muscles by about contraction 25 through the end of the ECC-induced injury protocol (Fig 5A and 5B at Day 0).

During recovery of strength in the following days, there was a significant main effect of group on isometric torque after ECC-induced injury (p = 0.006). OVX mice had a 13% lower recovery of isometric torque compared to sham mice (Fig 5B). There was also a main effect of days (p < 0.001), but no effect on their group × days interaction (p = 0.242). Sham female *mdx* mice recovered 103 ± 7% of pre-injury isometric torque by day 10, indicating full recovery of strength, whereas OVX female *mdx* mice recovered only 87 ± 5% by day 10. At day 14 and 21 d post-injury, isometric torque in OVX female *mdx* mice remained lower than their pre-injury isometric torque and sham female *mdx* mice. These results reveal that estrogen deficiency dampens the capacity of muscles to recover from ECC-induced injury in female *mdx* mice.

## Discussion

The purpose of this study was to investigate the impact of estrogen deficiency on skeletal muscle strength and the recovery of strength after injury in female *mdx* mice. Results from the first two experiments that examined ventilatory function and skeletal muscle contractile function do not support the hypothesis that deficits of estrogen decrease skeletal muscle strength in female *mdx* mice. However, the main finding in this study supports the hypothesis that estrogen deficiency diminishes the capacity of muscle to recover strength after a physiological injury in female *mdx* mice.

The present study demonstrates that estrogen impacts the capacity of muscle to recover from a physiological injury, as indicated by strength loss in OVX female *mdx* mice compared to sham female *mdx* mice during recovery after ECC-induced injury. The impairment was measured by the overall loss of torque during recovery leading to a blunted recovery of pre-injury isometric torque in OVX mice compared to sham mice. The concept that estradiol is the ovarian hormone protective against ECC-induced injury in *mdx* mice was also tested in a study by Hourde and colleagues [49]. They found a significant loss of strength between 6 month-old female *mdx* mice compared to 20 month-old female *mdx* mice after 9 ECC. However, they found no difference in strength loss between E2-treated and untreated 20 month-old female *mdx* mice after 9 ECC indicating that E2 is not protective to muscle when challenged simultaneously by aging and dystrophin deficiency [49]. Indeed, recovery of strength was impaired in OVX *mdx* mice relative to sham *mdx* mice in the current study, consistent with our previous work in wild-type (WT) mice demonstrating that OVX mice recovered ~20% less isometric torque 1–3 weeks after injury compared to sham mice or E2-treated OVX mice [34,35,50,51].

To recover from injury, skeletal muscle undergoes a regenerative process in order to restore damaged muscle fibers. Skeletal muscle regeneration following injury is dependent on resident muscle stem cells, termed satellite cells. Satellite cells upon a stimulus, such as an injury, are activated, proliferate, differentiate, and either fuse to each other to form new muscle fibers or to existing muscle fibers. We, among other labs, have shown that estrogen deficiency causes a decline in satellite cell number in muscles of female mice [50,52]. Few studies have investigated the impact of estrogen on regeneration in dystrophin-deficient muscle. Salimena et al. found in 6 week-old *mdx* mice, that OVX *mdx* mice had decreased regenerating fibers compared to control female *mdx* mice [53]. This decrease in regenerating fibers in OVX mice supports the notion that estrogen deficiency compromises the ability of skeletal muscles to functionally recover after injury in *mdx* and WT female mice [34,35,50].

Respiratory muscles, including the diaphragm, are vital in sustaining ventilation. Previous studies show that ventilatory function in *mdx* mice was compromised by low tidal volume, indicative of hypoventilation relative to wildtype mice [40,41,54]. Further, with age, *mdx* mice had greater diaphragm muscle atrophy and fibrosis, leading to increased respiratory impairment compared to WT mice [55–57]. Previous work assessing ventilatory function in *mdx* mice using WBP has been predominantly performed with males [58–60], with some studies pooling male and female mice [41,61], or with sex not reported [62]. This study is the first that we know of to report ventilatory function in only female *mdx* mice across ages. Our results suggest that estrogen deficiency had an insignificant influence on ventilatory function as deduced from a lack of difference between sham and OVX female *mdx* mice across all measures. Decreasing patterns were measured in most ventilatory parameters over time, which is consistent with previous literature on respiratory function in aging *mdx* mice [55,57]. Such declines in parameters such as respiratory rate and tidal volume during the HH challenge suggest that as *mdx* mice aged from 6 to 15 months, there may be an onset of respiratory impairment more likely attributed to the progression of disease rather than the loss of estrogen.

While estrogen deficiency did not affect skeletal muscle strength, fatigability, or recovery of strength after fatigue in both the diaphragm and anterior crural muscles of female *mdx* mice in this study, gender and age effects have been shown to impact skeletal muscle physiology of dystrophic mice [63,64]. For example, Hakim et al. showed that maximal sP_o_ of EDL muscle in 6 mo female *mdx* mice was greater than aged-matched male *mdx* mice [63]. Additionally, Hourde et. al. reported that in the TA muscle, 20 month-old female *mdx* mice (presumably estrogen deficient) had 21% lower maximal sPo compared to 6 mo female *mdx* mice, indicating that aging and/or loss of estrogen production was detrimental to muscle force generation [49]. In contrast, the present study indicates that estrogen deficiency alone does not affect skeletal muscle contractility in female *mdx* mice between the ages of ~6 to 15 months.

Diaphragm sP_o_ (~6 N/cm^2^) in this study was low compared to that reported by Beastrom et al. (~15 N/cm2) [65]. This difference is likely attributed to gender and age, as Beastrom et al. measured diaphragm force in 3 month-old male *mdx* mice compared to our 13–15 month-old (equivalent to human middle age of approximately 38–47 years-old) female *mdx* mice. Additionally, no difference in maximal sP_o_ by diaphragm muscle or maximal isometric torque of the anterior crural muscles was detected between sham and OVX mice. Indeed, there was no further worsening of ventilatory function or skeletal muscle contractile function in estrogen-deficient female *mdx* mice, suggesting that there may not be a strong physiological response to the loss of estrogen in the presence of a disease and/or without additional aging effects.

Manifesting carriers of DMD present with incongruent degrees of muscle weakness, pain, fatigue, and reduced endurance [9,66,67]. Although other *mdx* rodent models display stronger phenotypes similar to the human DMD disease, the *mdx* strain was selected because it is a genetic homolog to DMD and is the most studied and characterized strain since its discovery [68,69]. WBP was used to assess ventilatory function, as it is a non-invasive technique that can provide a multitude of functional information. However, as only ventilatory behaviors were assessed by WBP in this study, assessing higher‐force respiratory behaviors and near‐maximal expulsive behaviors necessary for airway clearance would be ideal to fully assess respiratory function. The gold standard to measure diaphragm muscle force across a range of respiratory behaviors, including higher-force respiratory behaviors is transdiaphragmatic pressure [70]. Therefore, whether these higher-force behaviors are compromised in estrogen-deficient female *mdx* mice remains to be determined.

## Conclusion

The key finding of this study is that estrogen deficiency affects the recovery of strength after injury in skeletal muscle of female *mdx* mice. Although no effect of estrogen deficiency was detected on ventilatory function or skeletal muscle strength, fatigability, or recovery of strength in the diaphragm or anterior crural muscles of female *mdx* mice, the lack of evidence for estrogen-related differences in the present study does not rule out potential estrogenic differences in later ages. Better understanding of how estrogen deficiency blunts the recovery of skeletal muscle strength after injury in female *mdx* mice may help in the development of therapies to streamline efforts in rehabilitation of manifesting females suffering from DMD symptomology.
